# Supplementary material for: A Likelihood Approach for Real-Time Calibration of Stochastic Compartmental Epidemic Models
Source: PLoS Comput Biol. 2017 Jan 17;13(1):e1005257. doi: 10.1371/journal.pcbi.1005257 (PMC5240920; doi:10.1371/journal.pcbi.1005257)
Supplement: S1 File — (TAR.GZ) [file pcbi.1005257.s014.tar.gz › HSPH_Online-SI-Revision/EnKF07/n100-severe/n100-severe_EnKF2_table-8.pdf]

1	newly	3 weeks	spec	2276.	1024.82	{85., 924.}	89.7583	0.772894	newly	3 weeks	cumul	4764.	5668.72	{2315., 5248.}	74.4601	0.220726	newly	5 weeks	spec	4385.	496.68	{2., 595.}	94.447	1.73635	newly	5 weeks	cumul	12 438.	6881.53	{2752., 5349.}	81.3653	0.508652	newly	58 weeks	cumul	64 638.	7933.09	{3007., 6875.}	89.7768	1.11139
2	newly	3 weeks	spec	951.	7382.1	{310., 11 836.}	698.467	0.822316	newly	3 weeks	cumul	1661.	65 574.	{26 360., 121 183.}	3849.79	1.53114	newly	5 weeks	spec	4438.	1960.04	{1., 4238.}	70.8576	1.13739	newly	5 weeks	cumul	8385.	71 237.1	{31 953., 123 678.}	751.56	0.886054	newly	65 weeks	cumul	54 065.	74 425.	{32 969., 124 979.}	57.8773	0.227719
3	newly	3 weeks	spec	788.	9745.28	{1446., 21 101.}	1148.5	0.9999	newly	3 weeks	cumul	1184.	47 370.3	{13 911., 91 871.}	3904.03	1.46332	newly	5 weeks	spec	4947.	4935.33	{39., 9989.}	67.528	0.630924	newly	5 weeks	cumul	8233.	59 199.1	{20 112., 111 456.}	628.032	0.818351	newly	66 weeks	cumul	68 234.	73 469.5	{23 016., 111 829.}	46.0933	0.270251
4	newly	3 weeks	spec	1410.	6765.19	{1025., 17 737.}	392.565	0.573301	newly	3 weeks	cumul	2503.	49 577.2	{5293., 119 144.}	1882.11	1.11255	newly	5 weeks	spec	4326.	1688.84	{94., 3725.}	68.3708	0.759649	newly	5 weeks	cumul	9460.	54 496.9	{7169., 123 162.}	484.875	0.644795	newly	64 weeks	cumul	66 493.	57 451.9	{13 396., 123 444.}	59.5907	0.358768
5	newly	3 weeks	spec	2748.	1062.75	{130., 923.}	89.0069	0.822909	newly	3 weeks	cumul	5863.	5913.48	{2288., 5271.}	77.7813	0.290854	newly	5 weeks	spec	4410.	506.58	{3., 610.}	94.0698	1.68635	newly	5 weeks	cumul	13 943.	7155.2	{2731., 5447.}	82.5129	0.549438	newly	58 weeks	cumul	66 068.	8228.74	{2999., 6893.}	89.371	1.10939
6	newly	3 weeks	spec	1016.	5924.32	{1123., 10 887.}	486.868	0.634777	newly	3 weeks	cumul	1815.	43 010.3	{12 192., 96 286.}	2270.47	1.22793	newly	5 weeks	spec	3575.	1623.01	{96., 3727.}	65.9354	0.695777	newly	5 weeks	cumul	7380.	47 594.9	{13 040., 108 094.}	548.478	0.699344	newly	64 weeks	cumul	66 876.	50 316.1	{14 082., 112 302.}	54.6157	0.345036
7	newly	3 weeks	spec	815.	6142.2	{263., 12 827.}	685.993	0.775432	newly	3 weeks	cumul	1311.	62 276.4	{18 518., 108 403.}	4653.97	1.56419	newly	5 weeks	spec	3565.	1505.64	{0., 4048.}	73.136	1.42406	newly	5 weeks	cumul	6614.	66 752.2	{18 562., 121 787.}	914.959	0.941981	newly	65 weeks	cumul	69 295.	69 651.7	{19 734., 123 866.}	44.6405	0.255628
8	newly	3 weeks	spec	1680.	415.44	{0., 533.}	95.2417	1.63705	newly	3 weeks	cumul	3165.	7869.92	{837., 18 280.}	196.415	0.427341	newly	5 weeks	spec	3946.	132.46	{0., 93.}	97.	2.88262	newly	5 weeks	cumul	9677.	8226.55	{883., 18 399.}	88.361	0.533051	newly	61 weeks	cumul	59 021.	8428.59	{891., 18 399.}	87.8234	1.17769
9	newly	3 weeks	spec	240.	9158.47	{463., 20 987.}	3719.14	1.38077	newly	3 weeks	cumul	369.	51 090.3	{15 939., 117 138.}	13 745.6	2.01626	newly	5 weeks	spec	1645.	4406.98	{10., 9741.}	207.042	0.849371	newly	5 weeks	cumul	2651.	61 841.5	{21 932., 119 192.}	2233.67	1.28016	newly	66 weeks	cumul	62 553.	73 851.9	{29 406., 124 005.}	51.5168	0.211314
10	newly	3 weeks	spec	1249.	1717.05	{131., 3092.}	161.01	0.651356	newly	3 weeks	cumul	2193.	7767.42	{1546., 11 304.}	277.177	0.275336	newly	5 weeks	spec	4136.	520.52	{11., 1274.}	91.913	1.59149	newly	5 weeks	cumul	8808.	9195.48	{1813., 15 095.}	123.957	0.568368	newly	59 weeks	cumul	62 587.	10 534.7	{1845., 41 714.}	86.5185	1.20711
11	newly	3 weeks	spec	804.	6138.29	{260., 12 722.}	697.69	0.803409	newly	3 weeks	cumul	1294.	59 194.5	{18 936., 110 439.}	4478.26	1.55717	newly	5 weeks	spec	3948.	1725.05	{1., 4134.}	74.8483	1.41976	newly	5 weeks	cumul	7140.	64 136.6	{18 936., 119 309.}	805.942	0.912249	newly	65 weeks	cumul	63 055.	66 475.4	{18 936., 123 815.}	49.5232	0.292588
12	newly	3 weeks	spec	2111.	1063.6	{78., 903.}	93.0261	0.76563	newly	3 weeks	cumul	4254.	6166.26	{2309., 5323.}	88.8162	0.206856	newly	5 weeks	spec	4443.	436.18	{0., 579.}	92.4961	1.8386	newly	5 weeks	cumul	11 913.	7283.47	{2747., 5422.}	83.8236	0.496913	newly	58 weeks	cumul	66 884.	8137.18	{3008., 6937.}	89.8529	1.12634
13	newly	3 weeks	spec	2345.	1069.57	{98., 1115.}	89.7394	0.772216	newly	3 weeks	cumul	5116.	5948.17	{2431., 5232.}	78.1573	0.24419	newly	5 weeks	spec	4572.	513.96	{2., 597.}	94.0954	1.69613	newly	5 weeks	cumul	13 208.	7196.58	{2859., 6129.}	82.4706	0.528859	newly	58 weeks	cumul	66 392.	8282.36	{3007., 7058.}	89.4041	1.11061
14	newly	3 weeks	spec	1956.	946.73	{56., 914.}	87.0752	0.739096	newly	3 weeks	cumul	4187.	5463.26	{2295., 5181.}	74.6105	0.191031	newly	5 weeks	spec	3466.	411.97	{1., 592.}	91.2074	1.66369	newly	5 weeks	cumul	10 276.	6493.59	{2723., 5312.}	78.3804	0.443011	newly	58 weeks	cumul	61 739.	7352.9	{2977., 6881.}	89.8696	1.10193
15	newly	3 weeks	spec	1396.	1863.23	{0., 1746.}	181.064	1.40454	newly	3 weeks	cumul	2586.	7937.	{883., 19 822.}	273.89	0.449679	newly	5 weeks	spec	3233.	794.43	{0., 320.}	102.495	2.44666	newly	5 weeks	cumul	7869.	10 077.	{883., 27 507.}	152.4	0.707191	newly	60 weeks	cumul	52 704.	11 415.3	{883., 31 306.}	89.3684	1.30354
16	newly	3 weeks	spec	1498.	2816.87	{163., 14 499.}	211.212	0.738929	newly	3 weeks	cumul	2968.	13 024.4	{1332., 66 792.}	380.679	0.37689	newly	5 weeks	spec	3246.	782.11	{19., 2179.}	88.9122	1.40427	newly	5 weeks	cumul	8486.	15 255.8	{1493., 74 649.}	187.913	0.58729	newly	59 weeks	cumul	63 449.	16 790.3	{1547., 75 852.}	84.7372	1.1469
17	newly	3 weeks	spec	711.	6696.53	{306., 11 791.}	860.305	0.849295	newly	3 weeks	cumul	1220.	65 986.5	{28 904., 110 553.}	5310.49	1.65842	newly	5 weeks	spec	2605.	1501.38	{0., 3735.}	68.099	1.18999	newly	5 weeks	cumul	5099.	70 583.7	{33 390., 121 686.}	1287.32	1.09374	newly	65 weeks	cumul	53 195.	72 715.1	{35 865., 122 983.}	56.8374	0.228782
18	newly	3 weeks	spec	1434.	2300.9	{2., 8268.}	173.766	1.33535	newly	3 weeks	cumul	2751.	40 247.	{7135., 101 796.}	1371.19	1.01829	newly	5 weeks	spec	4058.	675.8	{0., 2194.}	90.8635	2.69679	newly	5 weeks	cumul	9240.	42 065.2	{7361., 104 611.}	373.217	0.615437	newly	63 weeks	cumul	55 677.	43 419.9	{7411., 113 635.}	59.5475	0.444416
19	newly	3 weeks	spec	1846.	3424.38	{1., 8384.}	196.606	1.46977	newly	3 weeks	cumul	3207.	40 848.3	{7104., 92 085.}	1182.97	0.976088	newly	5 weeks	spec	5657.	986.91	{0., 3114.}	86.3396	2.65506	newly	5 weeks	cumul	12 216.	43 511.5	{7292., 105 161.}	278.802	0.558796	newly	63 weeks	cumul	62 777.	45 569.4	{7450., 116 703.}	58.2134	0.450307
20	newly	3 weeks	spec	1317.	251.37	{0., 512.}	90.8284	1.72135	newly	3 weeks	cumul	2412.	6241.19	{785., 12 264.}	202.128	0.449968	newly	5 weeks	spec	4511.	38.65	{0., 79.}	99.1432	3.10786	newly	5 weeks	cumul	9394.	6373.97	{880., 13 056.}	72.0316	0.52116	newly	61 weeks	cumul	61 521.	6416.53	{908., 13 228.}	89.7485	1.24492
21	newly	3 weeks	spec	780.	6623.4	{309., 12 902.}	769.826	0.794593	newly	3 weeks	cumul	1243.	60 837.2	{20 828., 108 367.}	4797.77	1.58261	newly	5 weeks	spec	3321.	1615.24	{2., 4092.}	69.7043	1.12278	newly	5 weeks	cumul	6292.	65 663.5	{20 834., 121 794.}	949.217	0.961596	newly	65 weeks	cumul	63 478.	68 671.9	{24 996., 124 395.}	46.2375	0.240125
22	newly	3 weeks	spec	720.	3783.5	{4., 8413.}	509.942	1.2377	newly	3 weeks	cumul	1304.	44 034.4	{7194., 117 653.}	3278.62	1.3566	newly	5 weeks	spec	2369.	1225.06	{0., 3209.}	96.4162	2.21016	newly	5 weeks	cumul	4863.	47 307.6	{7424., 120 341.}	877.62	0.848457	newly	63 weeks	cumul	55 662.	49 723.7	{7439., 120 517.}	63.9543	0.386816
23	newly	3 weeks	spec	325.	9679.24	{381., 19 916.}	2887.83	1.25472	newly	3 weeks	cumul	484.	45 856.7	{10 922., 91 968.}	9377.56	1.83517	newly	5 weeks	spec	3196.	5177.79	{4., 12 225.}	128.021	0.97983	newly	5 weeks	cumul	4636.	58 360.4	{12 653., 97 221.}	1166.61	1.03695	newly	67 weeks	cumul	66 380.	69 450.8	{14 066., 106 816.}	42.3476	0.283844
24	newly	3 weeks	spec	356.	4782.14	{257., 10 886.}	1259.13	0.936376	newly	3 weeks	cumul	524.	37 778.5	{4873., 94 405.}	7109.64	1.59542	newly	5 weeks	spec	2825.	3348.53	{0., 10 487.}	135.957	1.45756	newly	5 weeks	cumul	4283.	44 914.	{5792., 96 709.}	955.045	0.857816	newly	68 weeks	cumul	68 010.	57 586.	{6541., 97 952.}	48.3264	0.379312
25	newly	3 weeks	spec	331.	4902.57	{284., 11 148.}	1395.52	0.989017	newly	3 weeks	cumul	441.	36 795.1	{4943., 91 094.}	8243.56	1.67594	newly	5 weeks	spec	2373.	3359.21	{0., 10 926.}	153.2	1.35503	newly	5 weeks	cumul	3717.	43 999.2	{5811., 91 311.}	1087.31	0.900963	newly	68 weeks	cumul	58 819.	56 632.5	{7008., 97 996.}	57.3977	0.381708
26	newly	3 weeks	spec	1190.	1073.62	{278., 1745.}	69.1597	0.366726	newly	3 weeks	cumul	2010.	21 734.5	{8899., 49 264.}	983.478	0.890412	newly	5 weeks	spec	4173.	162.42	{6., 254.}	96.1078	2.06547	newly	5 weeks	cumul	8492.	22 260.4	{9037., 49 310.}	169.161	0.326631	newly	62 weeks	cumul	69 666.	22 573.7	{9048., 49 310.}	69.19	0.643866
27	newly	3 weeks	spec	974.	2457.49	{1., 8312.}	254.264	1.31337	newly	3 weeks	cumul	1673.	39 660.9	{7131., 106 921.}	2275.14	1.17193	newly	5 weeks	spec	3834.	702.77	{0., 2802.}	87.5381	2.59213	newly	5 weeks	cumul	7556.	41 590.1	{7377., 106 921.}	462.466	0.628	newly	63 weeks	cumul	62 217.	43 139.3	{7407., 114 797.}	64.4889	0.485268
28	newly	3 weeks	spec	2141.	1098.53	{255., 1763.}	62.0341	0.484663	newly	3 weeks	cumul	4227.	20 918.1	{8657., 68 029.}	395.663	0.548608	newly	5 weeks	spec	4410.	183.49	{6., 242.}	95.8392	1.96585	newly	5 weeks	cumul	11 858.	21 491.4	{8886., 68 360.}	100.256	0.216617	newly	62 weeks	cumul	54 529.	21 850.7	{8901., 68 371.}	70.8474	0.576403
29	newly	3 weeks	spec	1545.	2701.79	{3., 8277.}	188.222	1.44093	newly	3 weeks	cumul	2666.	38 754.	{7155., 88 475.}	1361.69	1.03433	newly	5 weeks	spec	5090.	865.31	{0., 3002.}	86.0646	2.75893	newly	5 weeks	cumul	10 609.	41 052.	{7384., 104 244.}	305.328	0.573793	newly	63 weeks	cumul	65 892.	42 872.8	{7421., 113 898.}	57.0812	0.453597
30	newly	3 weeks	spec	1626.	2488.17	{145., 14 422.}	183.819	0.763939	newly	3 weeks	cumul	3106.	11 712.9	{1316., 66 448.}	329.015	0.371678	newly	5 weeks	spec	3975.	663.62	{17., 2156.}	91.0269	1.47804	newly	5 weeks	cumul	9727.	13 635.1	{1459., 74 408.}	162.641	0.64773	newly	59 weeks	cumul	54 604.	15 181.6	{1524., 75 430.}	88.474	1.13229
31	newly	3 weeks	spec	312.	4853.53	{283., 10 956.}	1469.46	1.00104	newly	3 weeks	cumul	454.	37 607.1	{4917., 90 856.}	8183.51	1.67095	newly	5 weeks	spec	3043.	3362.74	{1., 11 824.}	130.064	1.41454	newly	5 weeks	cumul	4393.	44 792.8	{5797., 91 099.}	923.934	0.841246	newly	68 weeks	cumul	60 937.	57 210.9	{7042., 98 128.}	55.4125	0.381827
32	newly	3 weeks	spec	434.	9344.8	{305., 20 887.}	2064.85	1.19228	newly	3 weeks	cumul	710.	48 494.5	{12 930., 97 002.}	6731.6	1.66806	newly	5 weeks	spec	2775.	4639.14	{17., 10 570.}	112.728	0.687025	newly	5 weeks	cumul	4597.	59 740.4	{20 537., 111 744.}	1206.89	1.04044	newly	66 weeks	cumul	62 201.	73 231.8	{29 136., 124 063.}	48.2286	0.217876
33	newly	3 weeks	spec	975.	9622.86	{1442., 20 926.}	900.148	0.924389	newly	3 weeks	cumul	1516.	48 254.6	{15 860., 95 075.}	3083.02	1.40359	newly	5 weeks	spec	5236.	5036.38	{15., 10 698.}	61.733	0.676755	newly	5 weeks	cumul	8992.	60 247.	{22 631., 111 633.}	570.367	0.757702	newly	66 weeks	cumul	68 723.	74 948.3	{31 415., 113 025.}	43.3664	0.191823
34	newly	3 weeks	spec	1779.	764.56	{23., 1066.}	81.0455	0.775016	newly	3 weeks	cumul	3127.	4509.56	{2319., 5820.}	60.9671	0.163025	newly	5 weeks	spec	4645.	374.26	{0., 662.}	92.2428	1.92072	newly	5 weeks	cumul	10 816.	5413.61	{2762., 6262.}	68.1656	0.463243	newly	58 weeks	cumul	64 121.	6436.31	{2899., 7511.}	89.9622	1.1383
35	newly	3 weeks	spec	295.	8865.96	{401., 17 579.}	2911.56	1.25248	newly	3 weeks	cumul	455.	48 357.2	{10 592., 96 244.}	10 528.	1.8903	newly	5 weeks	spec	2408.	4572.65	{1., 10 305.}	159.682	1.04142	newly	5 weeks	cumul	3628.	59 367.4	{12 687., 96 657.}	1539.26	1.12117	newly	67 weeks	cumul	61 775.	70 415.9	{14 094., 104 311.}	45.9823	0.242303
36	newly	3 weeks	spec	489.	7485.23	{283., 17 249.}	1443.45	0.979192	newly	3 weeks	cumul	783.	56 776.7	{18 584., 100 169.}	7151.17	1.75199	newly	5 weeks	spec	2506.	2156.15	{5., 5284.}	85.5176	0.93048	newly	5 weeks	cumul	4350.	62 844.8	{18 779., 114 703.}	1347.61	1.0744	newly	65 weeks	cumul	66 421.	66 155.9	{18 884., 124 888.}	46.6735	0.253011
37	newly	3 weeks	spec	391.	10 047.7	{757., 19 159.}	2471.85	1.19506	newly	3 weeks	cumul	633.	54 647.5	{14 164., 102 153.}	8533.09	1.84016	newly	5 weeks	spec	3280.	5561.94	{3., 12 257.}	136.628	1.0471	newly	5 weeks	cumul	5202.	67 746.5	{22 038., 104 612.}	1202.69	1.05289	newly	67 weeks	cumul	56 370.	82 373.5	{31 276., 108 242.}	60.8378	0.231445
38	newly	3 weeks	spec	728.	6401.74	{282., 13 188.}	802.997	0.80566	newly	3 weeks	cumul	1154.	55 221.4	{18 708., 97 587.}	4687.11	1.57879	newly	5 weeks	spec	4350.	1804.49	{0., 4198.}	81.7607	1.38589	newly	5 weeks	cumul	7337.	60 346.6	{18 774., 111 021.}	727.255	0.854683	newly	65 weeks	cumul	69 743.	62 948.2	{18 774., 116 651.}	46.147	0.287122
39	newly	3 weeks	spec	1971.	1139.24	{274., 1849.}	62.6971	0.460888	newly	3 weeks	cumul	3791.	19 928.	{8625., 68 202.}	427.738	0.570209	newly	5 weeks	spec	4327.	204.64	{8., 260.}	95.2706	1.88094	newly	5 weeks	cumul	11 151.	20 565.2	{8915., 68 528.}	103.633	0.221425	newly	62 weeks	cumul	53 564.	20 874.9	{8928., 68 534.}	72.5006	0.600845
40	newly	3 weeks	spec	1025.	7436.25	{710., 15 489.}	641.761	0.749929	newly	3 weeks	cumul	1775.	59 216.5	{27 817., 109 198.}	3236.14	1.45442	newly	5 weeks	spec	3528.	2073.77	{5., 5362.}	76.3535	1.00646	newly	5 weeks	cumul	7401.	65 054.4	{31 197., 116 109.}	780.399	0.89138	newly	65 weeks	cumul	51 673.	68 564.9	{33 154., 122 775.}	52.6086	0.193351
41	newly	3 weeks	spec	676.	10 145.9	{736., 18 975.}	1405.58	0.978251	newly	3 weeks	cumul	1025.	54 360.4	{14 145., 102 053.}	5203.46	1.62662	newly	5 weeks	spec	4452.	5546.13	{3., 12 097.}	93.9818	0.999489	newly	5 weeks	cumul	7242.	67 461.2	{21 929., 104 710.}	833.026	0.912302	newly	67 weeks	cumul	64 974.	81 972.4	{30 721., 108 159.}	43.5838	0.19048
42	newly	3 weeks	spec	1593.	1452.88	{0., 1706.}	145.203	1.54828	newly	3 weeks	cumul	2901.	6841.46	{913., 15 918.}	212.78	0.431954	newly	5 weeks	spec	4138.	788.41	{0., 884.}	98.9935	2.54092	newly	5 weeks	cumul	9608.	8754.88	{913., 20 865.}	127.336	0.759972	newly	60 weeks	cumul	61 993.	10 436.3	{913., 27 576.}	89.6069	1.3804
43	newly	3 weeks	spec	1692.	2503.62	{142., 14 347.}	185.475	0.832587	newly	3 weeks	cumul	3552.	11 893.6	{1319., 66 112.}	297.678	0.401457	newly	5 weeks	spec	3812.	717.61	{10., 2138.}	91.1912	1.66275	newly	5 weeks	cumul	9984.	13 930.5	{1477., 74 074.}	160.897	0.643589	newly	59 weeks	cumul	48 969.	15 797.5	{1578., 75 200.}	89.9818	1.0921
44	newly	3 weeks	spec	459.	9007.84	{721., 18 691.}	1872.42	1.1698	newly	3 weeks	cumul	732.	48 561.9	{13 056., 96 918.}	6535.17	1.68184	newly	5 weeks	spec	2554.	4522.21	{43., 9858.}	120.428	0.679491	newly	5 weeks	cumul	4392.	59 448.7	{21 148., 111 386.}	1257.88	1.05788	newly	66 weeks	cumul	67 035.	72 310.2	{29 192., 119 269.}	44.2842	0.205763
45	newly	3 weeks	spec	956.	5846.93	{1121., 10 841.}	515.194	0.649161	newly	3 weeks	cumul	1721.	41 182.5	{12 037., 94 814.}	2293.85	1.22866	newly	5 weeks	spec	3513.	1688.6	{100., 3868.}	64.3046	0.670592	newly	5 weeks	cumul	7079.	45 879.2	{13 228., 108 010.}	551.724	0.697733	newly	64 weeks	cumul	64 288.	48 794.6	{13 939., 112 013.}	55.1762	0.34881
46	newly	3 weeks	spec	720.	2684.33	{3., 8488.}	355.26	1.08121	newly	3 weeks	cumul	1225.	40 275.3	{7139., 88 655.}	3188.92	1.33453	newly	5 weeks	spec	3211.	731.72	{0., 2865.}	85.8997	2.37129	newly	5 weeks	cumul	5975.	42 338.1	{7379., 105 464.}	614.689	0.715569	newly	63 weeks	cumul	66 403.	43 541.3	{7420., 114 718.}	60.8029	0.447566
47	newly	3 weeks	spec	2512.	1372.17	{129., 746.}	114.032	0.932284	newly	3 weeks	cumul	5499.	6757.81	{1318., 9434.}	130.448	0.456976	newly	5 weeks	spec	4726.	516.33	{12., 320.}	94.0713	1.76863	newly	5 weeks	cumul	13 753.	8092.3	{1470., 9517.}	106.017	0.737077	newly	59 weeks	cumul	63 771.	9438.46	{1523., 11 085.}	92.3407	1.29327
48	newly	3 weeks	spec	1312.	7761.22	{1184., 20 656.}	496.623	0.624547	newly	3 weeks	cumul	2348.	49 451.5	{12 098., 119 467.}	2007.04	1.16484	newly	5 weeks	spec	4095.	2033.57	{139., 3957.}	63.5402	0.632607	newly	5 weeks	cumul	8888.	55 322.1	{13 847., 122 736.}	527.298	0.678428	newly	64 weeks	cumul	66 105.	58 685.8	{14 140., 123 665.}	57.1861	0.332042
49	newly	3 weeks	spec	197.	4727.12	{258., 10 244.}	2310.06	1.14097	newly	3 weeks	cumul	276.	38 532.1	{4896., 94 405.}	13 860.9	1.89252	newly	5 weeks	spec	2370.	3144.64	{0., 10 066.}	149.899	1.47583	newly	5 weeks	cumul	3259.	45 310.3	{5740., 96 492.}	1294.24	0.965437	newly	68 weeks	cumul	66 569.	57 294.2	{6831., 98 695.}	49.7929	0.377766
50	newly	3 weeks	spec	580.	10 485.3	{732., 20 022.}	1716.17	1.11578	newly	3 weeks	cumul	912.	50 427.3	{14 097., 96 568.}	5429.31	1.64931	newly	5 weeks	spec	4132.	5628.81	{2., 12 259.}	98.9272	0.950834	newly	5 weeks	cumul	6745.	63 926.9	{15 682., 101 821.}	849.413	0.913119	newly	67 weeks	cumul	62 718.	76 140.5	{20 023., 108 382.}	46.0591	0.214908
